# Preference for ground cover when selecting burrow entrances in plateau pikas

**DOI:** 10.1002/ece3.11564

**Published:** 2024-06-18

**Authors:** Rui Zhang, Wei Liu

**Affiliations:** ^1^ College of Coastal Agricultural Sciences Guangdong Ocean University Zhanjiang China; ^2^ Northwest Institute of Plateau Biology Chinese Academy of Sciences Xining China

**Keywords:** alpine meadow, burrowing behavior, ground covers, *Ochotona curzoniae*, soil compaction

## Abstract

Burrow‐dwelling animals such as the plateau pika (*Ochotona curzoniae*) often seek sturdy entrances for their burrows, which can reduce the need for frequent maintenance. The toughness of the ground surface is often reinforced by the interweaving of plant roots and often varies with the root characteristics. To better understand ground cover preferences when selecting burrow entrances by plateau pikas, we investigated the ratios of different ground covers at the rear of the entrances, as well as their coverage and underlying soil compaction in an undegraded alpine meadow on the Qinghai‐Xizang Plateau. The results indicated a clear preference hierarchy of sedges > forbs > grass > bare soil. This distribution was aligned with the soil compaction hierarchy of the topsoil layer beneath each cover type. The sedge coverage was significantly negatively correlated with burrow density, suggesting that plateau pikas opt for sturdy entrances with a natural inclination toward energy conservation. However, there is consensus that the population density of plateau pikas often reaches its maximum on almost nonvegetated “black soil beaches.” We hypothesized that the survival benefits brought about by vegetation degradation would be higher than the maintenance costs of burrow entrances.

## Graphical abstract

Plateau pika has a clear preference hierarchy in the ground covers for their burrow entrances: sedge > forb > grass > bare soil. However, the population density of plateau pikas often reaches its maximum on almost nonvegetated “black soil beaches.” We hypothesize the reproductive benefits brought about by vegetation degradation are higher than the maintenance costs of burrow entrances.

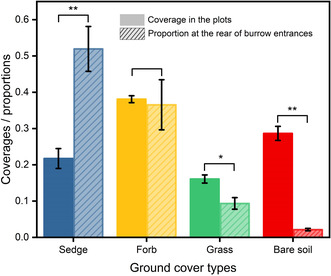

## 1 INTRODUCTION

Burrows with sturdy entrances can reduce the maintenance efforts of mammalian inhabitants. Compacted soil provides an ideal environment for construction; however, it often limits burrowing capabilities (Ducey et al., 1993). Burrowing in compact soil environments requires more energy (Vásquez, 2023). In the field, intensive digging activities are frequently correlated with soil softening (Ebensperger & Bozinovic, 2000). A sturdy ground cover paired with soft soil beneath it is optimal for burrow entrance construction, balancing stability and energy efficiency.

The interlacing of plant roots tends to bind to soil and enhance its integrity. In alpine meadows, plant roots typically create dense root mats near the soil surface (Kaiser et al., 2008). Plateau pikas (*Ochotona curzoniae*) often favor these areas for burrow entrances, which subsequently require minimal maintenance (Smith & Dobson, 2022). However, field observations indicated variability across the plateau landscape. Alpine meadows are not exclusively composed of *Kobresia* meadows. In particular, areas that have been overgrazed by livestock, which can be largely soil without sedges, appear to be favored by pikas, who reach their greatest density in these largely barren areas (Dong et al., 2013). In undegraded alpine meadows, although root mats provide excellent support for burrows, entrances are occasionally constructed on bare soil patches. Furthermore, root systems differ among plateau plant species; grasses have fibrous roots, forbs have taproots, and sedges have dense fibrous root systems (Liu et al., 2022; Miehe et al., 2019). Different root systems may cause differences in soil sturdiness, which may influence the preference of plateau pikas when constructing burrows.

Previous studies on burrow selection have primarily examined the impact of resource availability around nest sites (McMahon et al., 2017). However, in alpine meadows, for herbivorous species such as plateau pikas, which are extremely social (Smith & Dobson, 2022) and live in distinctly defined territories (in a family home range of approximately 24 m in diameter (Dobson et al., 1998)), differences in food resource acquisition are negligible, necessitating a focus on the energy costs related to burrow construction. For instance, research has shown that southern and western entrances are less inclined, which helps increase the temperature inside the burrow (Wei et al., 2013). Our study aimed to explore plateau pikas' selection of burrow covers, the vegetative environment in which to initiate burrowing, and the energy trade‐offs between excavation and maintenance.

Our research has implications for the management of alpine meadow systems, adaptive strategies for burrow‐dwelling species, and the construction of artificial burrows, enhancing the conservation of endangered burrowing animals.

## 2 MATERIALS AND METHODS

### 2.1 Site description

Our research was conducted in an undegraded *Kobresia pygmaea* alpine meadow in the northeastern Qinghai‐Xizang Plateau (37°58′ N, 100°14′ E, elevation 3660 m), China. The study site located in a flat winter pasture with no livestock during summer, which eliminated the impact of livestock trampling during the monitoring period. The average annual temperature was −3°C and the average annual precipitation was 420 mm.

### 2.2 Experimental design

**FIGURE 1:**
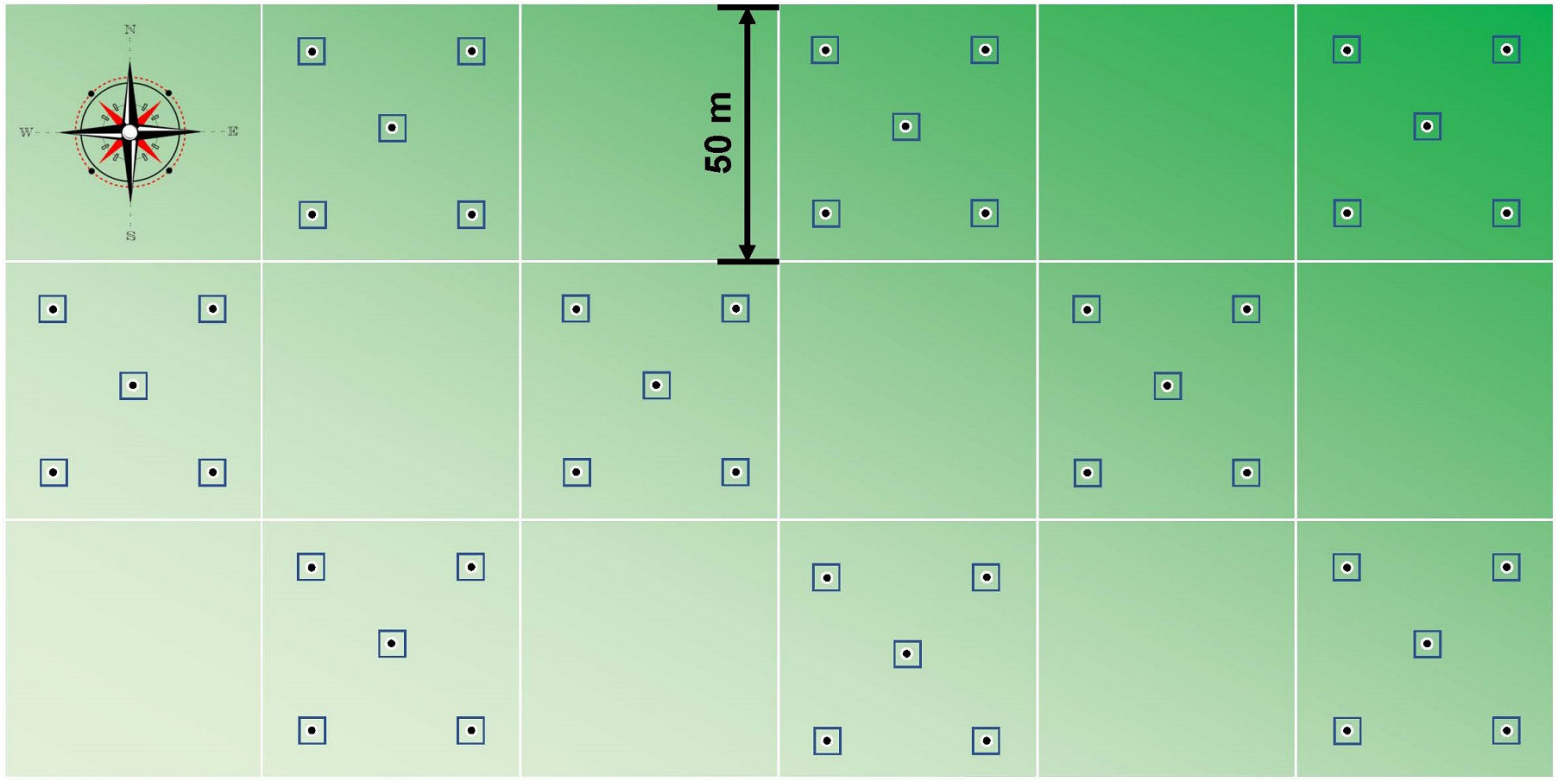
Schematic diagram of the sampling design. Solid dots represent soil compaction survey points, small boxes represent vegetation coverage survey plots, and color depth represents vegetation coverage.

Nine square plots, each measuring 50 × 50 m, were designated along the vegetation coverage gradient (Figure 1). The home range of a plateau pika family is 300–500 m^2^ (Qu et al., 2007; Smith & Dobson, 2022), indicating that each plot could contain to 5–8 pika families. Therefore, vegetation variation caused by the aggregation of plateau pikas can be minimized.

### 2.3 Field survey

**FIGURE 2:**
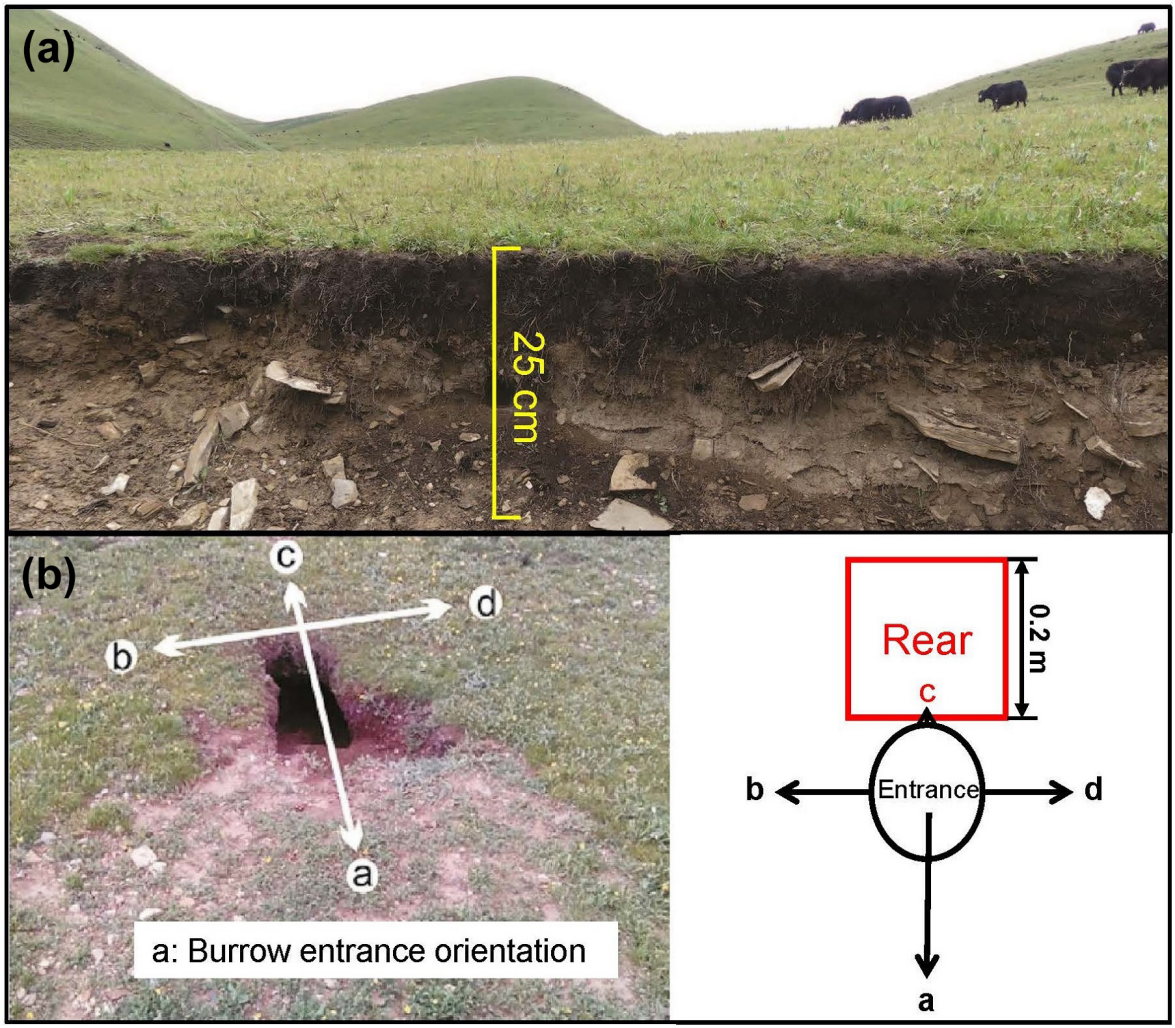
(a) Photos of the felty root mat of *Kobresia* (adapted from Smith et al., 2019), and (b) plateau pika burrow and schematic diagram of the rear of the burrow entrance (adapted from Zhang et al., 2016).

Monthly soil compaction surveys were conducted between April and September 2016. We performed measurements for at least 3 days following rainfall to eliminate the impact of soil moisture. Soil compaction was measured using a Spectrum SC‐900 soil compactor, with a range of 0 to 7000 kilopascal (kPa), ±103 kPa accuracy, and depth accuracy of ±1.25 cm. Given that plateau pikas often dig to depths between 20 and 50 cm (Wei et al., 2007), sampling was taken at 2.5 cm increments down to a depth of 25 cm (Figure 2a), until impeded or encountered burrow tunnels. Quincunx (Figure 1) and random sampling were used to investigate soil compaction in the bare soil and vegetation patches, respectively. All the selected patches were located more than 1 m away from the burrow entrance. A total of 5–10 repeats were selected for each vegetation cover type in each survey.

In mid‐August 2016, vegetation cover was surveyed based on quincunx sampling (Figure 1), with a quadrat size of 0.5 m × 0.5 m. Alpine meadow vegetation is usually divided into four functional groups of sedges, weeds, legumes, and grasses, respectively (Guo, Li, et al., 2012a; Zhang et al., 2016). Because legumes are mostly scattered in weeds and most have similar root systems, legumes and weeds were merged into forbs (Liu et al., 2022; Symstad, 2000). These three plant functional groups (sedge, forb, and grass) and bare soil constituted the four ground cover types in this study. The coverage of these ground covers was measured using the point‐grid method (Booth & Cox, 2008; Chen et al., 2007) with 25 point‐grids.

A plateau pika burrow entrance can be divided into three components: the entrance, the ejecta soil mound in front of the entrance, and the cover in the other three directions of the entrance. The firmness of the entrance was primarily influenced by the cover above the tunnel, which was the ground cover type at the rear of the burrow entrances (Figure 2b). The active state of the burrows was determined visually. Smooth tunnel walls, fresh grass blades, and fresh soil mounds in front of the burrow entrances were the distinguishing features (Zhang, Xu, & Liu, 2018b).

### 2.4 Ground cover favor index

The Ground cover favor index (FI) was calculated using Equation (1).

(1) $$\mathrm{FI}=R_{b}/R_{c}$$

where FI is the favor index, *R*_*b*_ is the ratio of burrow entrances to cover type, and *R*_*c*_ is the coverage ratio of burrow type in the plot.

FI > 1 indicates favor, FI < 1 indicates aversion. The higher the FI value, the higher the level of favor.

### 2.5 Statistical analysis

A Chi‐square test of independence was used to compare the burrow entrance covers and random availability of these ground cover types. By mixing the time‐series data, a linear mixed‐effects model was used to evaluate the soil compaction differences under different ground cover types. The significance threshold was set at *p* < .05, and all statistical analyses were performed using SPSS (version 23.0; SPSS Inc., Chicago, IL, USA).

## 3 RESULTS

### 3.1 Ground cover preferences

**FIGURE 3:**
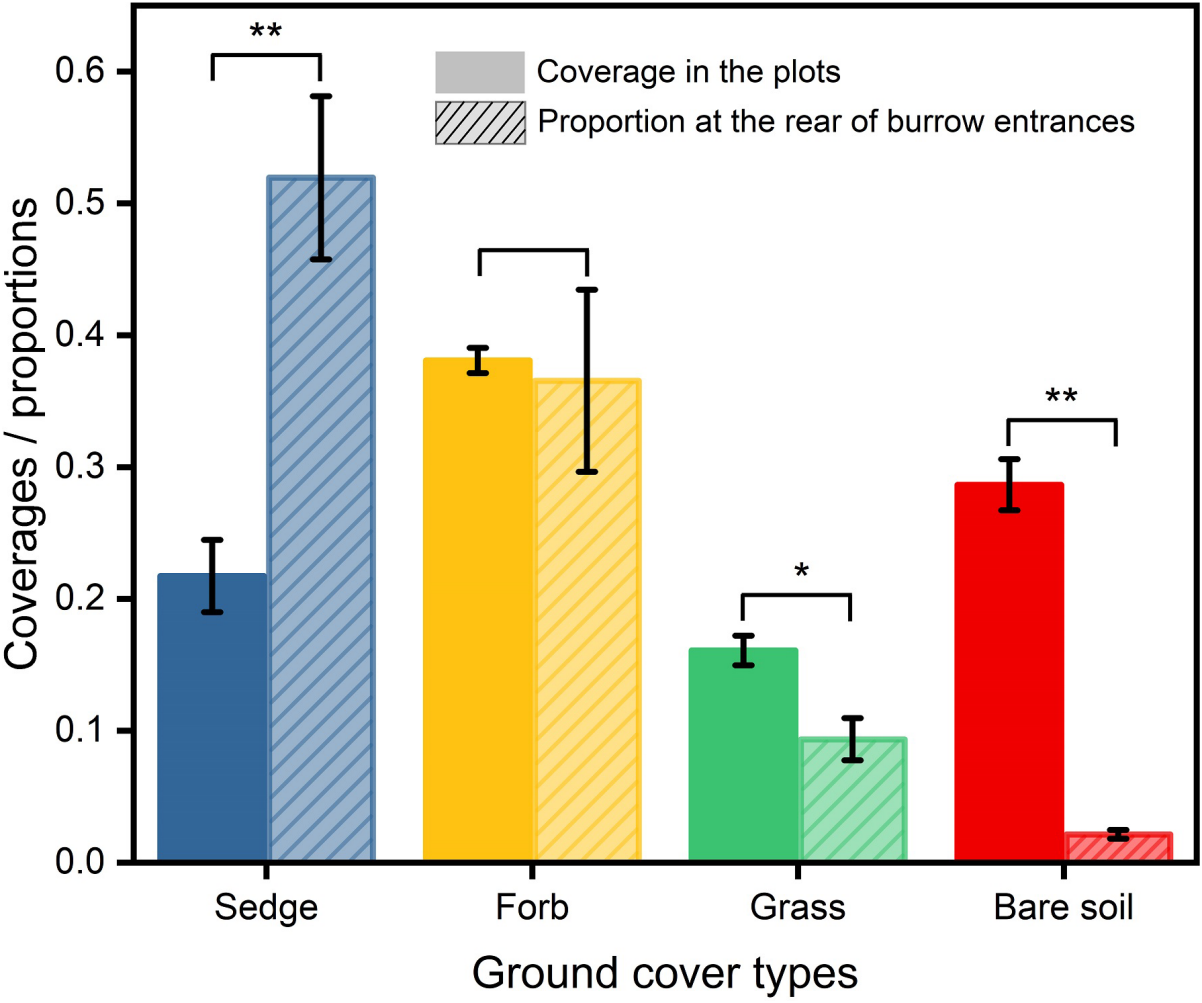
Coverages of sedge, forb, grass, and bare soil in the plots versus their proportions at the rear of the burrow entrances. The results are presented as the mean ± SE. * indicates a significant difference (*p* < .05), ** indicates a very significant difference (*p* < .01).

A total of 3693 active burrows were observed in the plots. At the rear of the burrow entrances, sedge patches accounted for 51.2%, forb patches for 37.3%, grass patches for 9.3%, and bare soil patches for 2.2%. In contrast, these cover types represented 20.3%, 35.9%, 15.1%, and 28.7% of coverage in the plots, respectively (Figure 3). Significant differences were observed in the proportion of ground cover types at the rear of the burrow entrances and their coverage in the sample plots (*p* < .01).

**FIGURE 4:**
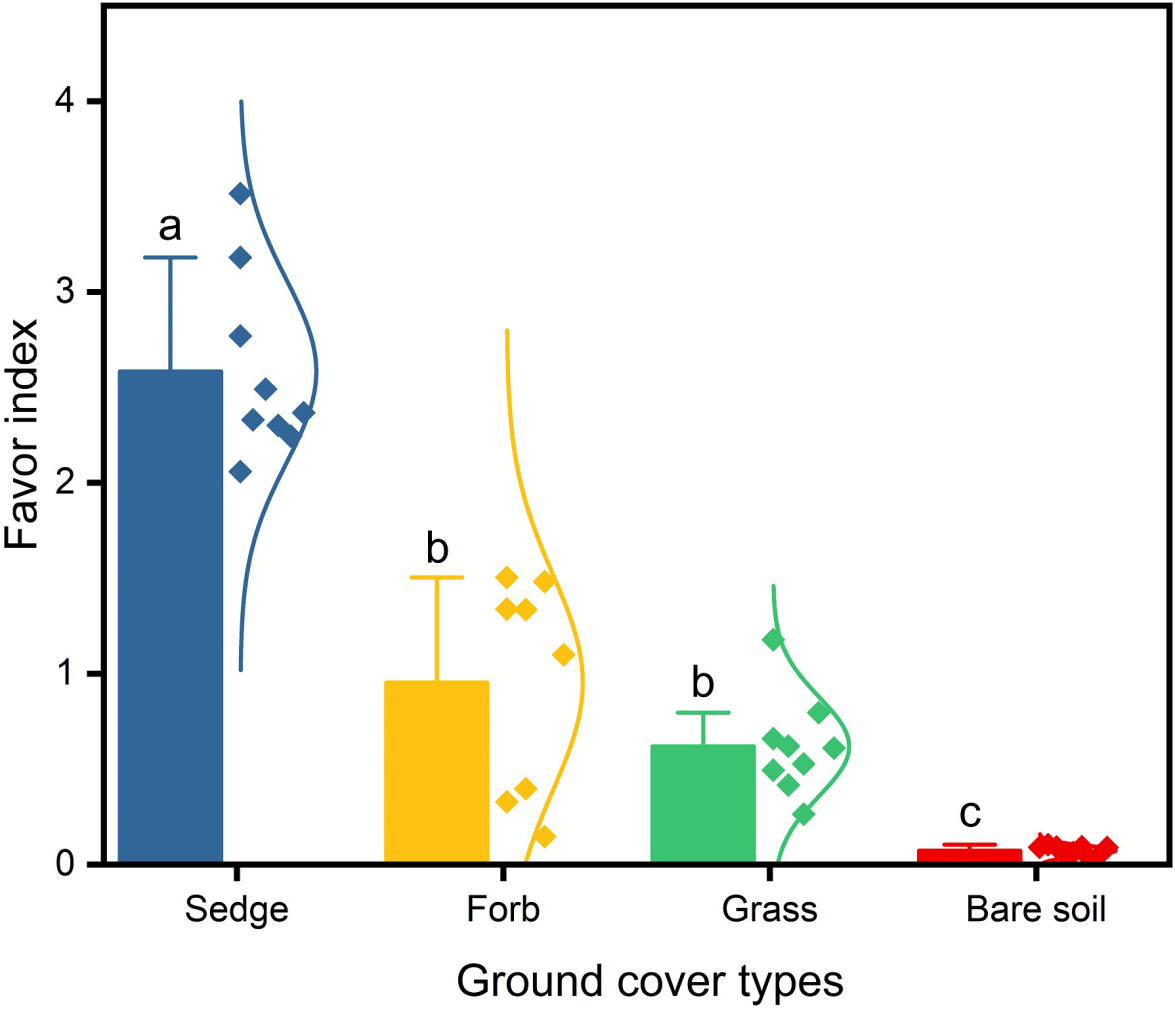
Favor indices for sedge, forb, grass, and bare soil. The results are presented as mean ± SE. Different letters indicate significant differences, with a significance level of *p* < .05.

Plateau pikas showed distinct favors for ground cover types at the rear of burrow entrances, with average favors indices for sedge at 2.58, forb at 1.00, grass at 0.62, and bare soil at 0.07 (Figure 4). The favorable differences were significant (*p* < .05) between ground cover types, except for the favorable differences between grasses and forbs. Favor indices skewed to one side of equivalency for all cover types, except for forbs, indicating ambiguity in preference for forbs.

### 3.2 Soil compaction varies with depth and cover type

**FIGURE 5:**
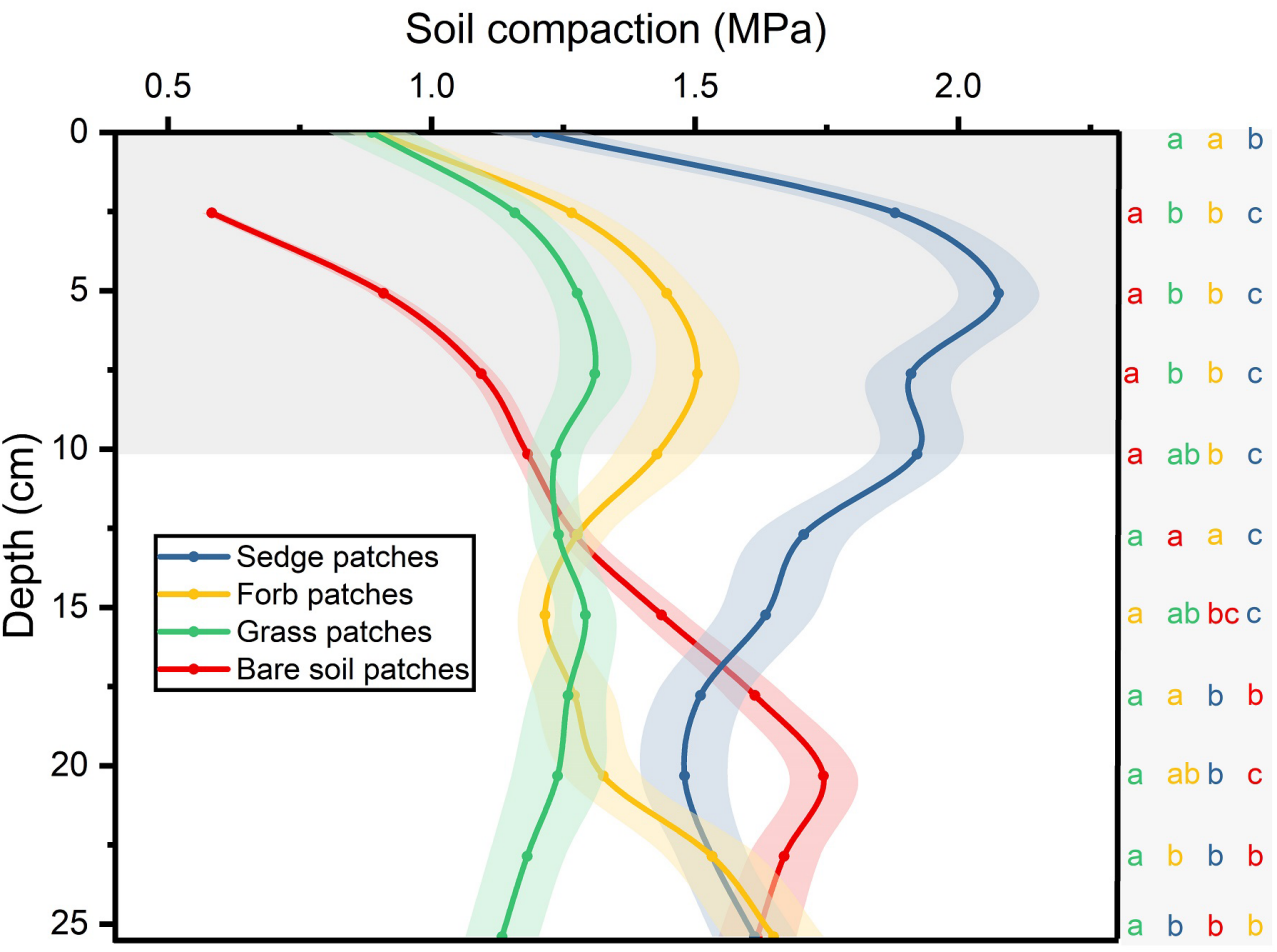
Soil compaction changes with depth under diverse cover types. The unit ‘MPa’ means megapascal. The results are presented as mean ± SE. Different letters indicate significant differences among ground covers in the same depth (*p* < .05).

Soil compaction varied significantly according to depth and cover type (*p* < .01) (Figure 5). Soil compaction was greatest under sedges at depths less than 10 cm, followed by forbs, grass, and bare soil. Beyond 20 cm, grass showed significantly lower compaction than the other soil types (*p* < .05). Compaction trends in sedges and forbs were similar, initially rising, then falling, before rising again, peaking at 25 cm for forbs and approximately 5 cm for sedges. Grass exhibited a modest increase, followed by a decrease, whereas bare soil increased steadily until the depth exceeded 20 cm and became relatively stable.

### 3.3 The variation of burrow density with coverages

**FIGURE 6:**
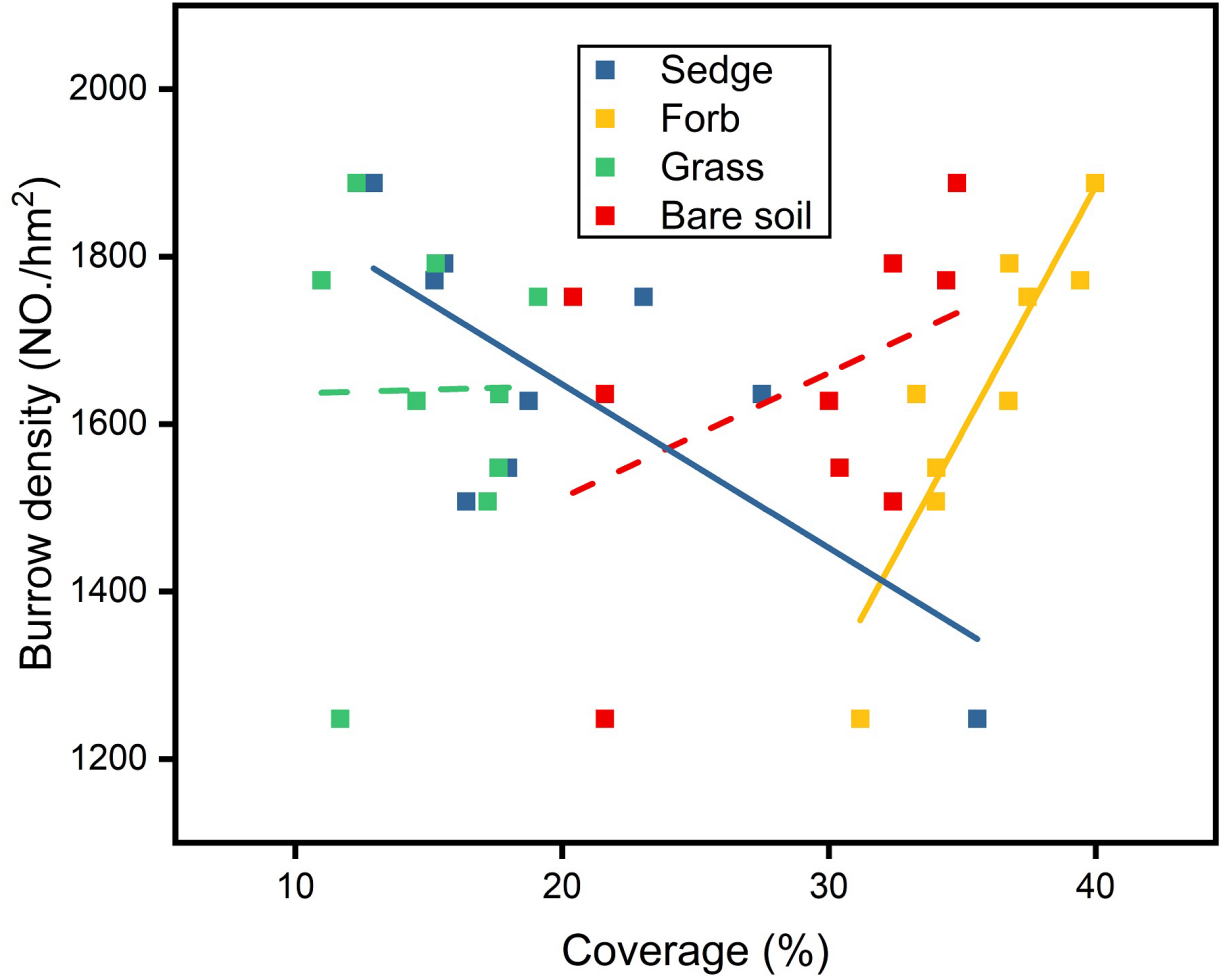
Burrow density changes with the coverage of sedge, forb, grass and bare soil. Solid lines indicate significance (*p* < .05), dash lines indicate no significance.

The relationship between the coverage of different ground covers and burrow density varied significantly (Figure 6). Sedge coverage was significantly negatively correlated with burrow density (*r* ^2^ = 0.55, *p* < .05), whereas forb coverage was significantly positively correlated with burrow density (*r* ^2^ = 0.81, *p* < .05). There was no significant correlation between the coverage of grass and burrow density, and the relationship was not apparent. A positive correlation existed between bare soil coverage and burrow density, but this relationship was not significant.

## 4 DISCUSSION

According to the results, plateau pikas exhibited a preference for particular cover types in the choice of burrow sites. Two competing scenarios may explain these observations: (1) plateau pikas actively and preferentially select sedge meadow vegetation to construct their burrow entrances and (2) they potentially burrow randomly but do not maintain entrances that become unsuitable. These two scenarios correspond to the construction of two types of burrows. There are two distinct types of burrows—deep ones connected within a social family territory and shallow, unconnected “duck” holes which may constitute up to a third of the burrows in a family territory (Dobson et al., 1998). Given that pikas expand family burrows from the inside to maintain tunnel connectivity and reduce predation, burrow entrances on the surface should be unrelated to the ground cover, matching the prevalence of ground cover. We hypothesize this selectivity may occur through the abandonment of fragile entrances, including those often collapsed by livestock trampling (Holmes et al., 2003; Šklíba et al., 2017), rain erosion (Hayne, 1936), or other specific conditions (e.g., severe cold or pregnancy) that lead to blockage of the burrow (Zhou et al., 2023), which is a passive selection process. For the “duck” holes, plateau pikas have to burrow from the ground surface. The meadow surface contains “edges” which are preferentially used by pikas—often for initiating burrows (Paruchuri et al., 2019), which is an active selection on ground covers. It is worth noting that, there may also exist abandonment of “duck” holes. The observed discrepancy in proportions and burrowing strategies suggests both active and passive selectivity toward the ground cover.

The selection by plateau pikas appears to be correlated with soil compaction near the surface of the ground, aligning with the pikas' preference for burrow cover. Based on the principle of random sampling, the probability of this being a coincidence was exceedingly low ($\frac{1}{4!}=4.2\%$). Although the higher the soil compaction, the higher the initial cost of excavation, given that burrows are semipermanent, with some being abandoned and reused (Liu et al., 2013; Pech et al., 2007), sturdy entrances may reduce maintenance costs for multiple generations. The negative correlation between sedge vegetation coverage and burrow density observed in the present study reflects a decrease in energy expenditure during sturdy entrances. This discovery has implications for the adaptive strategies of burrow‐dwelling species and the design of artificial burrows that can help conserve endangered burrowing animals.

Variations in soil compaction under different cover types may be attributed to their root structures. Sedges possess short, fibrous, and dense roots (Miehe et al., 2019; Raab et al., 1996) which often form solid and almost impenetrable felty root mats (Kaiser et al., 2008; Smith et al., 2019). Forbs in alpine meadows mostly feature taproots (Liu et al., 2022), and often colonize bare ground on mounds of earth that cover the dominant sedge (*Kobresia*) meadow (Liu et al., 2012). There may still be a large number of undecomposed sedge roots in the soil, which gives some of them a certain degree of toughness. Fibrous root grasses are often scattered in alpine meadows (Liu et al., 2022), which makes it difficult for them to form a dense root network. Bare soil typically results from the prolonged absence of plant growth, where any remaining root systems may decompose and lose their toughness.

Although plateau pikas prefer to construct burrow entrances under strong vegetation, they adapt well to vegetation degradation. The density of plateau pikas often peaks on large, exposed “black soil beaches” (Dong et al., 2013). Given that bare soil is not the preferred cover type for plateau pikas, living on heavily degraded meadows inevitably increases the costs associated with maintaining burrow entrances. Therefore, we hypothesized that the survival benefits of vegetation degradation would be higher than the maintenance costs of burrow entrances. These benefits may be due to a higher survival rate in the presence of predators. Plateau pikas rely heavily on vision to detect predators (Wei et al., 2022). In degraded alpine meadows with fewer plants, plateau pikas have a better vision for detecting predators, thereby reducing the probability of predation (Mao et al., 2024; Wang et al., 2024). Prioritizing the reduction in vegetation coverage is also a strategy chosen by plateau pikas for territorial expansion (Zhang, Liu, & Xu, 2018a). Assessing the intensity and implications of these changes in maintenance efforts requires extensive, long‐term research.

The vegetation on the Qinghai‐Xizang Plateau has undergone significant changes (Miehe et al., 2019). Owing to global warming, the Qinghai‐Xizang Plateau has gradually become arid (Gao et al., 2015; Xue et al., 2017). Deeper plants such as forbs and grasses, which have greater drought tolerance (You et al., 2014), are replacing shorter‐rooted sedges in alpine meadows (Liu et al., 2018; Mou et al., 2022). Furthermore, additional factors, such as overgrazing, identified as drivers of alpine meadow degradation (Harris, 2010), contribute to the increasing presence of bare soil patches (Liu et al., 2003). Therefore, research on the adaptation mechanism of burrowing behavior to vegetation change will have long‐term and broad significance. This is particularly important for the conservation of endangered burrowing animals sensitive to environmental changes.

Plateau pikas serve as ecosystem engineers in the alpine meadow ecosystem of the Qinghai‐Xizang Plateau (Hogan, 2010). Burrow systems not only provide shelters for breeding, shelters from severe cold, and refuges from predators, but also serve as habitats for numerous animals, such as birds and insects, after abandonment (Foggin & Smith, 1996; Smith & Foggin, 1999). A reduction in plateau pika burrow density can significantly diminish bird diversity and abundance (Lai & Smith, 2003; Arthur et al., 2008). Moderate‐density burrows can increase water infiltration, reduce surface runoff, and contribute positively to soil conservation (Wilson & Smith, 2015). Physically, their digging activities enhance soil permeability, oxygen content (Martin, 2003), water conductivity (Ma et al., 2015), and water‐holding capacity (Guo, Zhou, & Hou, 2012b), while decreasing soil bulk density, compaction, and thermal conductivity (Ma et al., 2015). Moderate digging activities can also increase soil organic carbon content (Pang et al., 2015) and facilitate soil exchange between different depths, thereby enhancing nitrogen and mineral cycling in the soil (Sun et al., 2015; Zhang et al., 2016). Considering the favor of plateau pikas for ground cover in selecting burrow entrances, regulating their digging behavior through vegetation management may be a new way to maintain the ecosystem function of alpine meadows.

## 5 CONCLUSION

Plateau pikas prefer robust and tightly rooted ground cover for burrow entrances to save energy in the order of sedge, forb, grass, and bare soil, which correlates with near‐surface soil compaction. This preference can result from either the active selection of sites to construct burrows or the abandonment of flimsy entrances.

## AUTHOR CONTRIBUTIONS

**Rui Zhang:** Conceptualization (lead); data curation (lead); formal analysis (lead); funding acquisition (equal); investigation (lead); methodology (lead); project administration (supporting); resources (equal); software (lead); supervision (supporting); validation (lead); visualization (lead); writing – original draft (lead); writing – review and editing (lead). **Wei Liu:** Funding acquisition (equal); project administration (lead); resources (equal); supervision (lead).

## FUNDING INFORMATION

This work was supported by the Program for Scientific Research Start‐up funds of Guangdong Ocean University (060302052322) and the National Natural Science Foundation of China (30970498).

## CONFLICT OF INTEREST STATEMENT

The authors declare no competing interests.

## Data Availability

The datasets generated during and/or analyzed during the current study are available on the Baidu Netdisk at https://pan.baidu.com/s/1FgCufQ0I7VX11OsXmrNOGg?pwd=d6is.
